# Comparative efficacy and safety of Xuebijing injection as adjuvant therapy in sepsis-associated acute kidney injury: a systematic review and meta-analysis 

**DOI:** 10.3389/fphar.2025.1643557

**Published:** 2025-10-21

**Authors:** Bofei Shu, Xu Zhou, Jing Fan, Can Yang, Wei Deng, Bangjiang Fang, Huan Zhang

**Affiliations:** ^1^ Graduate School, Jiangxi University of Chinese Medicine, Nanchang, China; ^2^ Evidence-Based Medicine Center, Jiangxi University of Chinese Medicine, Nanchang, China; ^3^ Department of Radiology, The First Affiliated Hospital, Jiangxi Medical College, Nanchang University, Nanchang, China; ^4^ Institute of Emergency and Critical Care Medicine, Shanghai University of Traditional Chinese Medicine & Longhua Hospital, Shanghai, China; ^5^ Nursing Department, The Affiliated Hospital of Jiangxi University of Chinese Medicine, Nanchang, China

**Keywords:** Xuebijing injection, sepsis, sepsis-associated acute kidney injury, traditional Chinese medicine, systemic review

## Abstract

**Background:**

Xuebijing injection is a standardized traditional Chinese medicine formulation comprising extracts from safflower, red peony, Chuanxiong, Angelica, and Salvia miltiorrhiza. It is clinically employed for the treatment of sepsis and associated complications.

**Methods:**

This systematic review evaluated the efficacy and safety of Xuebijing injection in treating sepsis-associated acute kidney injury (SA-AKI). Six databases were searched up to 1 September 2024, to identify randomized controlled trials (RCTs) comparing Xuebijing injection combined with conventional therapies versus the same conventional therapies alone. Data from individual RCTs were synthesized by meta-analysis, with effect measures expressed as risk ratios (RRs) or mean differences (MDs) and their 95% confidence intervals (CIs). Trial sequential analysis was used to assess the precision of the effect estimates, and the GRADE system was used to evaluate the quality of evidence.

**Results:**

Eighteen RCTs involving 1,650 patients were included. Meta-analysis demonstrated that, compared with conventional therapies alone, Xuebijing injection combined with conventional therapies significantly reduced 28-day mortality (RR 0.82%, 95% CI 0.69–0.98). It also significantly improved renal function (serum creatinine level: MD -17.55 μmol/L, 95% CI: −23.22 to −11.88; blood urea nitrogen level: MD -1.58 mmol/L, 95% CI -1.83 to −1.32; urine volume: MD 5.83 ml, 95% CI: 3.45–8.21), inflammatory cytokines (tumor necrosis factor-alpha level: MD -29.20 ng/ml, 95% CI: −39.15 to −19.25; interleukin-6 level: MD -25.80 ng/mL, 95% CI: −35.56 to −16.04; interleukin-10 level: MD -8.02 ng/mL, 95% CI: −13.98 to 2.07), and immune function (percentage of CD3^+^ T cells: MD 10.30%, 95% CI 7.77%–12.84%; percentage of CD3^+^ T cells: MD 9.57%, 95% CI 3.53%–15.61%; CD4/CD8 ratio: MD 0.27, 95% CI 0.18–0.36). In addition, Xuebijing injection significantly alleviated the severity of SA-AKI as measured by the Acute Physiology and Chronic Health Evaluation II score (MD -3.12, 95% CI: −4.51 to −1.73). Subgroup analyses suggested potential effect modifications based on treatment duration or dosage. All reported adverse reactions were mild.

**Conclusion:**

Xuebijing injection may help reduce mortality and improve renal function in patients with SA-AKI. However, the certainty of evidence ranged from moderate to very low, underscoring the need for validation through large-scale, double-blind randomized controlled trials.

## 1 Introduction

Sepsis is a life-threatening organ dysfunction caused by a dysregulated host response to infection, contributing to an estimated 5.3 million global deaths annually (Evans et al., 2021; Fleischmann et al., 2016). The harm not only comes from uncontrolled infection and systemic inflammation but also from complications that accelerate deterioration and increase mortality. Sepsis-associated acute kidney injury (SA-AKI) is a frequent complication among patients with sepsis. A prospective cohort study across 24 European countries revealed that the incidence of AKI in sepsis ranged from 30% to 50%, with a greater incidence observed in severe sepsis or septic shock cases (Vincent et al., 2006). Epidemiological studies from the United States and China indicate that SA-AKI occurs in approximately 20%–25% of all septic patients admitted to intensive care units, highlighting its substantial clinical burden (Wang et al., 2021; Takeuchi et al., 2025). Recent prognostic tools such as the LIP score, which integrates lymphocyte count, INR, and procalcitonin, highlight progress in sepsis risk stratification (Liu et al., 2022). Nevertheless, therapeutic strategies specifically targeting SA-AKI remain scarce. The mortality among patients with SA-AKI is significantly greater than those without kidney injury, reaching up to 41% (Pais et al., 2024). Clinically, SA-AKI often manifests as oliguria or anuria, accompanied by refractory metabolic acidosis and fluid overload, which exacerbates prognosis. Beyond the systemic inflammatory response caused by sepsis, SA-AKI involves renal injury mechanisms, including renal tubular epithelial cell damage, a decreased glomerular filtration rate, changes in renal hemodynamics, and excessive activation of inflammatory mediators within the kidney (White et al., 2023). Therefore, SA-AKI not only poses an immediate threat to patient survival but is also associated with long-term renal dysfunction and chronic kidney disease, with some survivors progressing to end-stage renal disease requiring lifelong dialysis or transplantation (Vijayan et al., 2021).

Currently, except for supportive care and timely renal replacement therapy, there is no specific treatment for SA-AKI. The primary therapeutic objectives involve preventing further renal impairment by optimizing hemodynamics and avoiding nephrotoxic agents through fluid resuscitation and anti-infection strategies (Pickkers et al., 2021). However, fluid resuscitation in patients with renal insufficiency may exacerbate fluid load, potentially worsening renal function. Studies indicate that continuous renal replacement therapy utilizing blood purification techniques, can rapidly remove inflammatory mediators from the systemic circulation, thereby attenuating renal tubular epithelial cell injury. However, there is still a lack of internationally treatment regimens for blood purification, resulting in application that often relies on local clinical expertise (Zou et al., 2022). In addition, the high cost of blood purification restricts its utilization in areas with low economic levels (Taha et al., 2024). Therefore, there is a need to investigate more cost-effective therapeutic interventions for SA-AKI.

In China, the therapeutic potential of traditional Chinese medicine in managing sepsis and its complications has garnered growing interest. Xuebijing injection, launched in 2004, is the only standardized traditional Chinese medicine formulation approved by the Chinese National Health Commission for treating sepsis and systemic inflammatory response syndrome, and it was approved for severe COVID-19 in 2020 (Hu, 2023). Mechanistically, SA-AKI involves a dual pathological process: an uncontrolled systemic inflammatory response—often termed a “cytokine storm”, marked by excessive release of TNF-α, IL-6, and IL-10—coexists with immune suppression, characterized by T cell dysfunction and reduced immune surveillance. This imbalance contributes to endothelial injury, microcirculatory dysfunction, and progressive renal parenchymal damage. Key metabolites in Xuebijing, including hydroxysafflor yellow A, paeoniflorin, ligustrazine, salvianolic acids, and ferulic acid, have demonstrated strong binding affinity to core targets within the NF-κB and related signaling pathways. Experimental investigations indicate that these metabolites not only suppress the overproduction of pro-inflammatory cytokines but also enhance T-cell-mediated immunity, improve microcirculatory perfusion, and preserve residual renal function. These pharmacological effects directly target the inflammation-immune dysregulation axis implicated in SA-AKI (Chen et al., 2023).

Since its introduction into clinical practice, Xuebijing injection has been utilized in the management of SA-AKI, and multiple randomized controlled trials (RCTs) have been conducted to evaluate its efficacy. However, the results across these trials are inconsistent and often limited by small sample sizes and inadequate statistical power. Previous systematic reviews have assessed the effects of Xuebijing injection on sepsis in general rather than specifically focusing on SA-AKI, or have assessed its role in non-septic AKI; no systematic reviews have exclusively focused on the efficacy and safety of Xuebijing injection in SA-AKI (Zheng et al., 2018; Li et al., 2023). Moreover, existing systematic reviews exhibit methodological shortcomings, including frequent absence of protocol registration, suboptimal risk-of-bias assessments, and limited appraisal of quality of evidence. Additionally, the safety profile of Xuebijing injection specifically in SA-AKI patients remains unassessed. Therefore, we conduct this systematic review of RCTs to investigate both the efficacy and safety of Xuebijing injection in SA-AKI, aiming to provide comprehensive evidence for its clinical application in this specific condition.

## 2 Materials and methods

### 2.1 Description of the intervention

This study complies with the four pillars of best practice in ethnopharmacology regarding pharmacognostic characterization, pharmacological relevance, clinical safety, and contextual relevance.

Xuebijing injection is a standardized traditional Chinese medicine formulation manufactured by Tianjin Hongri Pharmaceutical Co., Ltd. It is approved by the National Medical Products Administration of China (registration number Z20040033). In April 2020, a supplementary approval (approval number 2020B02811) was issued to update its labeling to include the indication for “severe and critically ill COVID-19 patients with systemic inflammatory response syndrome and/or multiple organ dysfunction syndrome.” The product standard follows YBZ01242004-2010Z-2012. Quality control procedures for Xuebijing injection involve the identification and quantification of key bioactive compounds, including hydroxysafflor yellow A, danshensu, ferulic acid, ligustrazine, and paeoniflorin. Previous reviews indicate that Xuebijing injection exhibits a favorable safety profile, with no major adverse events reported (Sun et al., 2015).

All botanical names were validated using the Medicinal Plant Names Services (MPNS) database. Pharmacopoeial drug names and standards were confirmed against with the Pharmacopoeia of the People’s Republic of China (2020 Edition).

### 2.2 Study profile

The study protocol was prospectively registered on the PROSPERO platform (registration number: CRD42024521450). This systematic review was conducted and reported in accordance with the 2020 Preferred Reporting Items for Systematic Reviews and Meta-Analyses (PRISMA) guidelines (PRISMA) (Page et al., 2021).

### 2.3 Eligible criteria (PICO framework)

#### 2.3.1 Population (P)

RCTs involving adult patients (≥18 years) diagnosed with SA-AKI were eligible. The diagnosis of SA-AKI adhered to established guidelines, typically defined as kidney injury occurring within 24 h after sepsis diagnosis, characterized by either an increase in serum creatinine of ≥0.3 mg/dL (≥26.5 μmol/L) within 48 h, a rise to ≥1.5 times the baseline value persisting for over 7 days, or a reduction in urine output to <0.5 mL/kg/h for more than 6 h. Studies investigating AKI of non-septic etiology (e.g., acute tubular necrosis, glomerulonephritis, interstitial nephritis, or postrenal obstruction) were excluded.

#### 2.3.2 Intervention (I)

RCTs administering Xuebijing injection as an adjunctive therapy to conventional treatment were eligible. No restrictions were applied to dose or treatment duration. As Xuebijing injection is exclusively manufactured by Tianjin Hongri Pharmaceutical Co., Ltd. in a single dosage form in China, the preparation was presumed consistent across studies. Accordingly, all trials reporting the use of Xuebijing injection from this manufacturer were incorporated, irrespective of whether explicit compositional details were provided. Studies combining Xuebijing injection with other traditional Chinese medicines were excluded.

#### 2.3.3 Comparator (C)

RCTs comparing the combination of Xuebijing injection and conventional therapies against conventional therapies alone are eligible. Conventional therapies can be antibiotics, glucocorticoids, vasoactive drugs, fluid resuscitation, nutritional support, blood purification, and other interventions aligned with guideline recommendations. Studies comparing Xuebijing injection combined with one conventional therapy against an alternative conventional therapy were excluded.

#### 2.3.4 Outcomes (O)

The primary outcomes were 28-day all-cause mortality and the incidence of adverse events. The 28-day all-cause mortality was selected as a primary outcome due to the high mortality rate associated with SA-AKI and because short-term survival is the most widely accepted and objective endpoint in sepsis trials. This outcome aimed to capture the most clinically significant benefit of Xuebijing injection on patient prognosis. The incidence of adverse events was also designated as a primary outcome to comprehensively assess the safety profile of Xuebijing injection, given that ensuring drug safety is paramount in critically ill populations.

Secondary outcomes included renal function indicators (serum creatinine, blood urea nitrogen, and urine volume), inflammatory cytokines (TNF-α, IL-6, IL-10), immune function parameters (percentage of CD3^+^ T cells, percentage of CD4^+^ T cells, CD4+/CD8+ ratio), and severity of critical illness as assessed by the Acute Physiology and Chronic Health Evaluation II (APACHE II) score. These secondary outcomes were chosen to reflect intermediate physiological effects and potential mechanisms, which may serve as surrogate indicators in studies where primary outcomes were unreported, thus providing supplementary clinical insights.

### 2.4 Literature search

We searched six electronic literature databases to identify relevant studies: PubMed, Embase, China National Knowledge Infrastructure, Wanfang Data, VIP, and the China Biomedical Database. The search period extended from the inception of each database to 1 September 2024. Key search terms used in the search strategies included intervention-related (e.g., “Xuebijing injection,” “Xue Bi Jing injection”) and condition-specific phrases (e.g., “sepsis-associated acute kidney injury”, “SA-AKI”). Detailed search strategies are provided in Supplementary Table S1. In addition, we manually searched the reference lists of relevant reviews to identify potentially omitted studies. The bibliographies obtained from the search were imported into Endnote X9.3.3 software for management.

### 2.5 Screening and data extraction

Two reviewers independently screened the records and extracted data in accordance with the predefined eligibility criteria. The initial screening phase involved the exclusion of clearly irrelevant studies based on title and abstract evaluation. Subsequently, the full texts of potentially eligible studies were assessed to confirm their adherence to all eligibility criteria. Data were extracted from each included study using a pre-designed data extraction form, capturing information such as the first author’s name, publication year, sample size, patient age, details of intervention and control regimens, outcome data, and methodological elements essential for risk of bias assessment. In cases of incomplete or ambiguous data, efforts were made to contact the original authors for clarification. Any discrepancies between reviewers during screening or extraction were resolved through discussion or, when necessary, arbitration by a third reviewer.

### 2.6 Risk of bias assessment

The risk of bias for each included study was independently assessed by two reviewers using the Cochrane Risk of Bias tool, version 2 (RoB 2) (Sterne et al., 2019). This tool covers five domains of potential bias, namely, bias during the randomization process, bias due to deviation from the intended intervention, bias in outcome measurement, bias due to incomplete outcome data, and bias due to selective reporting of results. A study was classified as having an overall “low risk of bias” only if all domains were judged as at low risk. Conversely, if one or more domains were assessed as having “some concerns” or being at “high risk,” the overall risk of bias was categorized accordingly, indicating either some concerns or a high risk of bias. The reviewers cross-verified all assessments, and any disagreements were resolved through discussion or, when consensus could not be reached, by arbitration from a third reviewer.

### 2.7 Statistical analysis

Meta-analysis was performed to synthesize data from individual RCTs for the efficacy outcomes. For binary outcomes, the relative risk (RR) with 95% confidence interval (CI) were used as the effect size, and the effects were combined using the Mantel‒Haenszel method. For the continuous outcomes, the mean difference (MD) with 95% CI was employed as the effect measure, and pooling was conducted using the inverse variance method. We evaluated the possibility of false-positive or false-negative errors in the meta-analytic results by trial sequential analysis (TSA) (Kulinskaya and Wood, 2014), with the type I error probability set at 0.05, the power at 0.80, and the traditional Z threshold of 1.96. In addition, to assess the impact of the risk of bias on the robustness of the results, we performed sensitivity analysis with the exclusion of studies with an overall high risk of bias.

Statistical heterogeneity was assessed using Cochran’s Q test and the I^2^ statistic. Heterogeneity was considered significant if the p value of the Q test was <0.10 or I^2^ was ≥50%. A fixed-effect model was applied in the absence of significant heterogeneity; otherwise, a random-effects model was used. For the outcomes with significant heterogeneity, we performed subgroup analysis to explore potential sources of heterogeneity. The factors used for stratification included the dose of Xuebijing injection (100 mL/d vs. 200 mL/d), treatment duration (≤7 days vs. >7 days) and mean age of the experimental group (≤50 years vs. > 50 years). For the outcomes involved in 10 or more RCTs, funnel plots and Egger’s test were used to detect whether there was significant publication bias. All statistical analyses were conducted using RevMan (version 5.4), TSA software (version 0.9.5.10 Beta), and R (version 4.3.3).

### 2.8 Quality of evidence appraisal

The Grading of Recommendations, Assessment, Development and Evaluation (GRADE) system was used to assess the quality of evidence for all outcomes (Balshem et al., 2011). Since the meta-analyses were based on RCTs, the initial level of evidence was high. For the outcomes with limitations across the five GRADE domains, namely, risk of bias, inconsistency, indirectness, imprecision and publication bias, the quality of evidence was downgraded to moderate, low, or very low.

## 3 Results

### 3.1 Results of the literature search

**FIGURE 1:**
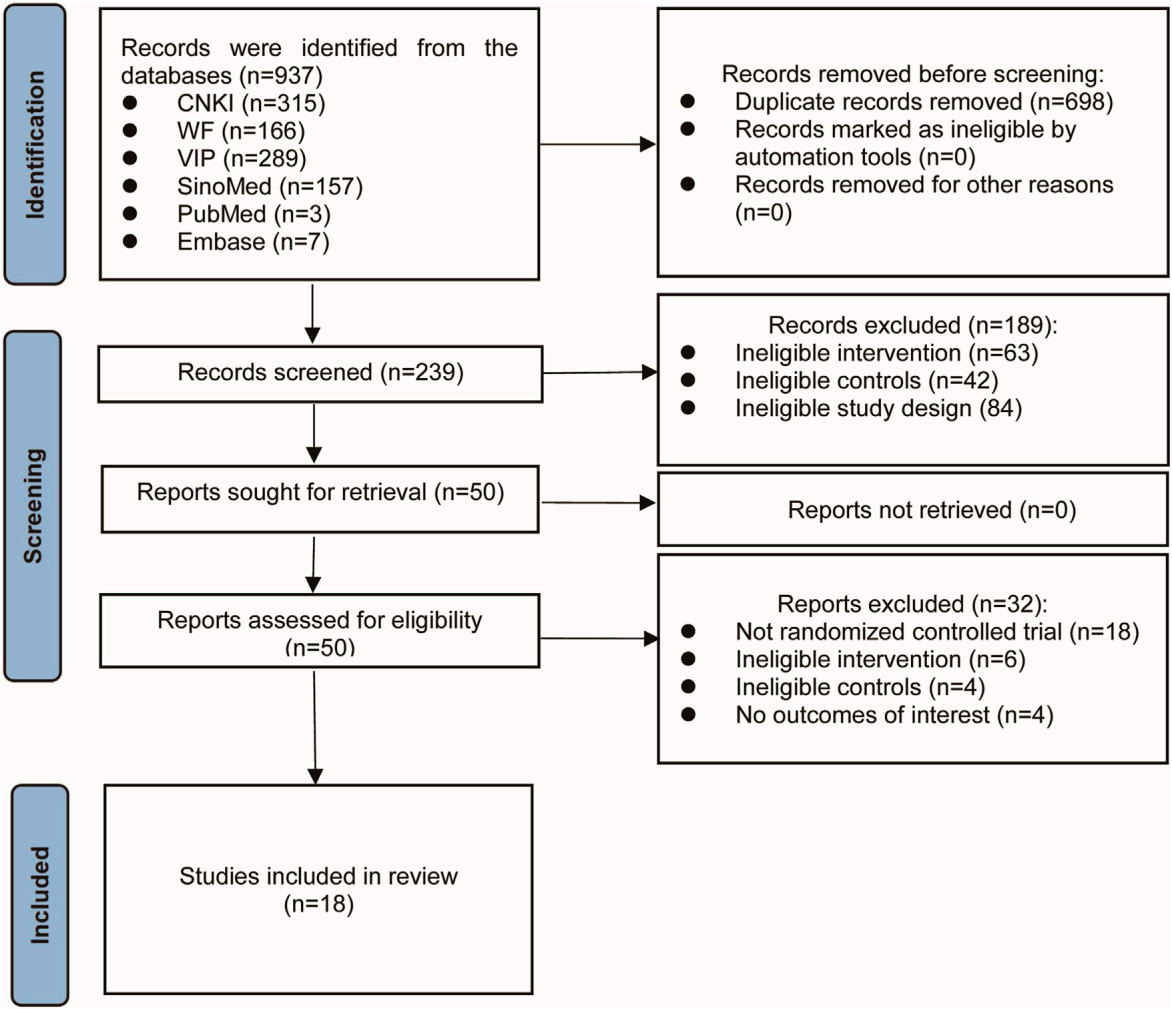
Process of literature screening.

A total of 937 records were initially identified through systematic searches. After removing duplicates and screening titles and abstracts, 50 studies remained for further evaluation. Following a full-text review of these studies, 18 eligible RCTs were ultimately identified (Tang et al., 2014; Lin et al., 2014; Zhang et al., 2016; Yang, 2016; Jiang et al., 2017; Wan et al., 2018; Luo et al., 2018; Zhang S. Z. et al., 2019; Zhang M. et al., 2019; Li et al., 2019; Huang, 2019; Liu et al., 2020; Zhu and Wu, 2020; Zhao, 2020; Yu, 2021; Huang et al., 2021; Song et al., 2022; Lin et al., 2022). Figure 1 shows the detailed process of study selection. The main excluded publications and reasons for exclusion are shown in Supplementary Table S2.

### 3.2 Characteristics of the included studies

The 18 included RCTs enrolled a total of 1,650 patients, with individual study sample sizes ranging from 38 to 292. The average age of the patients across studies ranged from 38 to 68 years. In the Xuebijing injection group, there were 460 males and 368 females; in the control group, there were 449 males and 373 females. All trials reported using Xuebijing injection manufactured exclusively by Tianjin Hongri Pharmaceutical Co., Ltd. The intervention of Xuebijing injection was administered intravenously in all studies; it was diluted in 0.9% sodium chloride solution in 14 trials and in 5% glucose solution in the remaining four trials. The administered dosage was 50 mL twice daily in 7 trials and 100 mL twice daily in 11 trials. The treatment duration was ≤7 days in 12 trials and >7 days in 6 trials. Concurrent continuous renal replacement therapy was applied in 13 trials. Detailed characteristics of the included RCTs are presented in Table 1.

**TABLE 1:** Taxonomical and pharmacopoeial details of the botanical drugs used in Xuebijing injection.

Chinese drug name	Botanical name (with authority)	Family	Pharmacopoeial drug name
Honghua	Carthamus tinctorius L	Asteraceae	Carthami Flos
Chishao	Paeonia lactiflora Pall	Paeoniaceae	Paeoniae Radix Rubra
Chuanxiong	Ligusticum chuanxiong Hort	Apiaceae	Chuanxiong Rhizoma
Danggui	Angelica sinensis (Oliv.) Diels	Apiaceae	Angelicae Sinensis Radix
Danshen	Salvia miltiorrhiza Bunge	Lamiaceae	Salviae miltiorrhizae Radix et Rhizoma

### 3.3 Risk of bias

**FIGURE 2:**
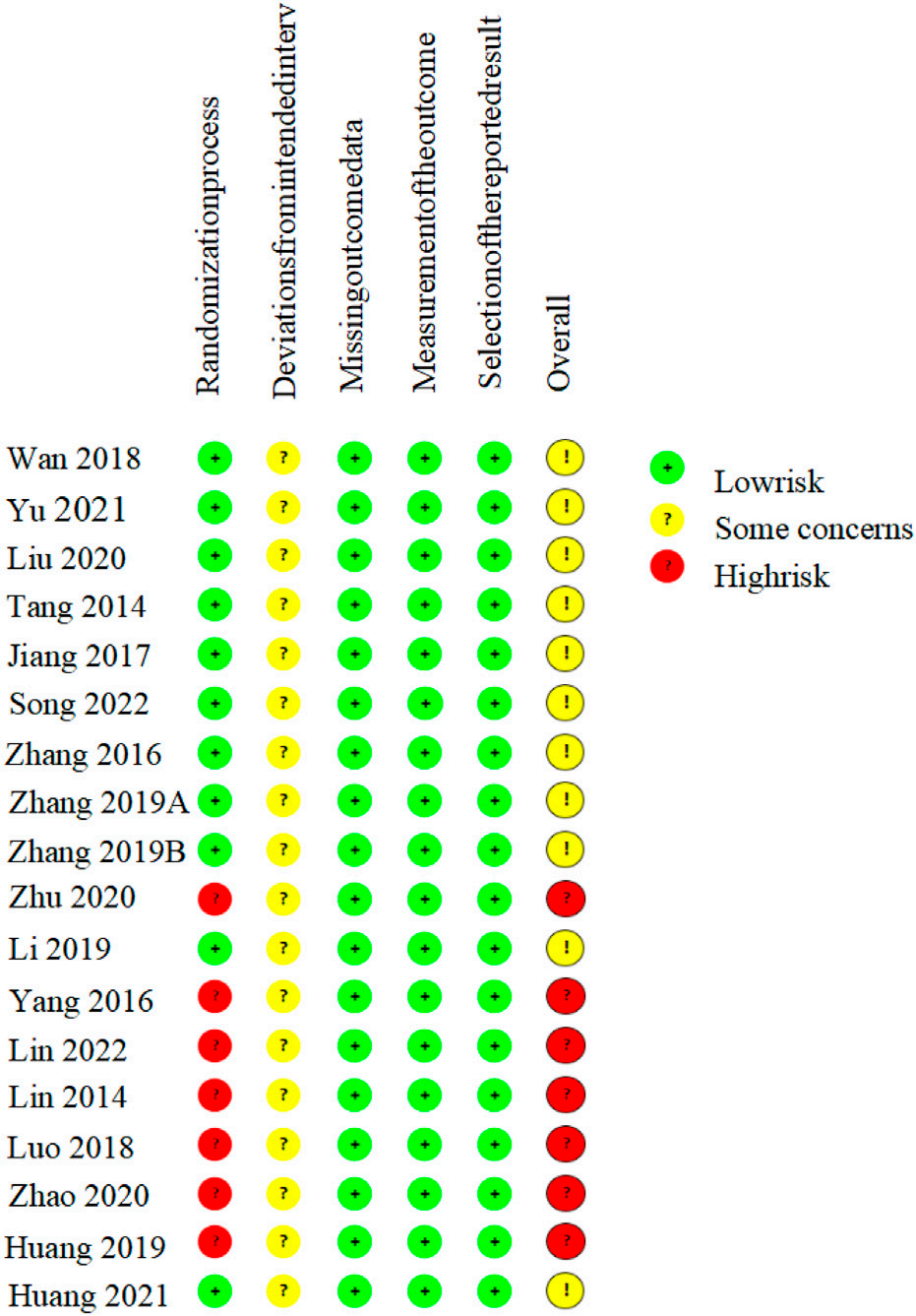
Results of the risk of bias assessment.

All 18 included RCTs reported the use of randomization, of which 11 (Tang et al., 2014; Zhang et al., 2016; Jiang et al., 2017; Wan et al., 2018; Zhang S. Z. et al., 2019; Zhang M. et al., 2019; Li et al., 2019; Liu et al., 2020; Yu, 2021; Huang et al., 2021; Song et al., 2022) specified using a random number table for random sequence generation; the remaining studies did not report specific methods for generating random sequences. All studies did not disclose whether allocation concealment or blinding was implemented. There was no evidence of selective reporting or significant attrition bias in any included trial. The absence of allocation concealment and blinding raises concerns regarding potential performance and detection biases, which may have led to overestimation of the treatment effects. Overall, seven RCTs were rated as having a moderate risk of bias, and 11 were judged to have a high risk of bias (Figure 2).

### 3.4 28-day mortality

**FIGURE 3:**
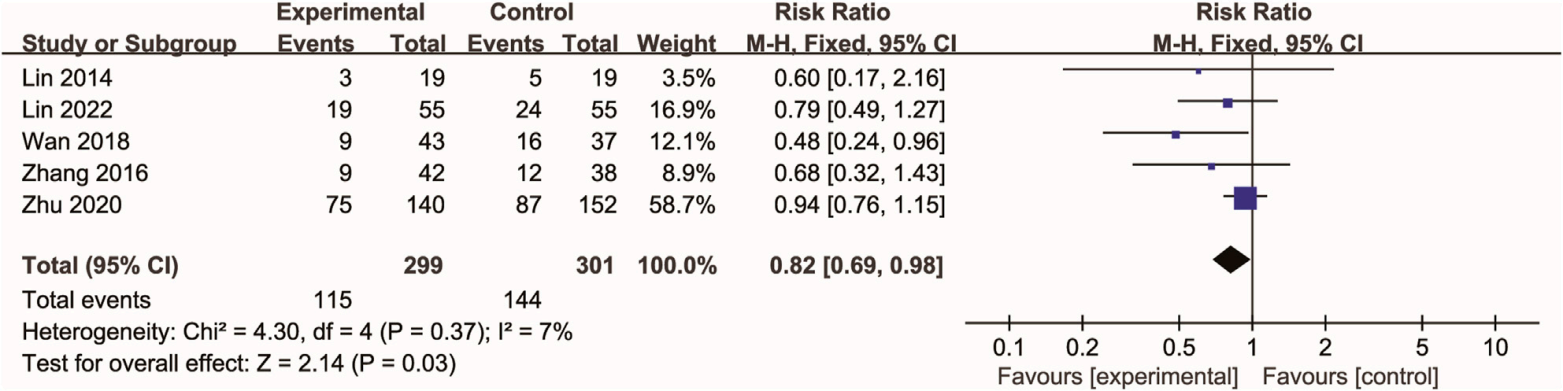
Forest plot of the meta-analysis of 28-day mortality.

Five RCTs (Lin et al., 2014; Zhang et al., 2016; Wan et al., 2018; Zhu and Wu, 2020; Lin et al., 2022) evaluated 28-day mortality. In the Xuebijing injection group, 115 deaths (38.46%) were recorded, while the control group experienced 144 deaths (47.84%). As shown in Figure 3, meta-analysis demonstrated a statistically significant reduction in 28-day mortality in the Xuebijing injection group compared to the control group (RR 0.82, 95% CI 0.69 to 0.98; p = 0.03). There was no significant heterogeneity among the RCTs.

### 3.5 Kidney function

**FIGURE 4:**
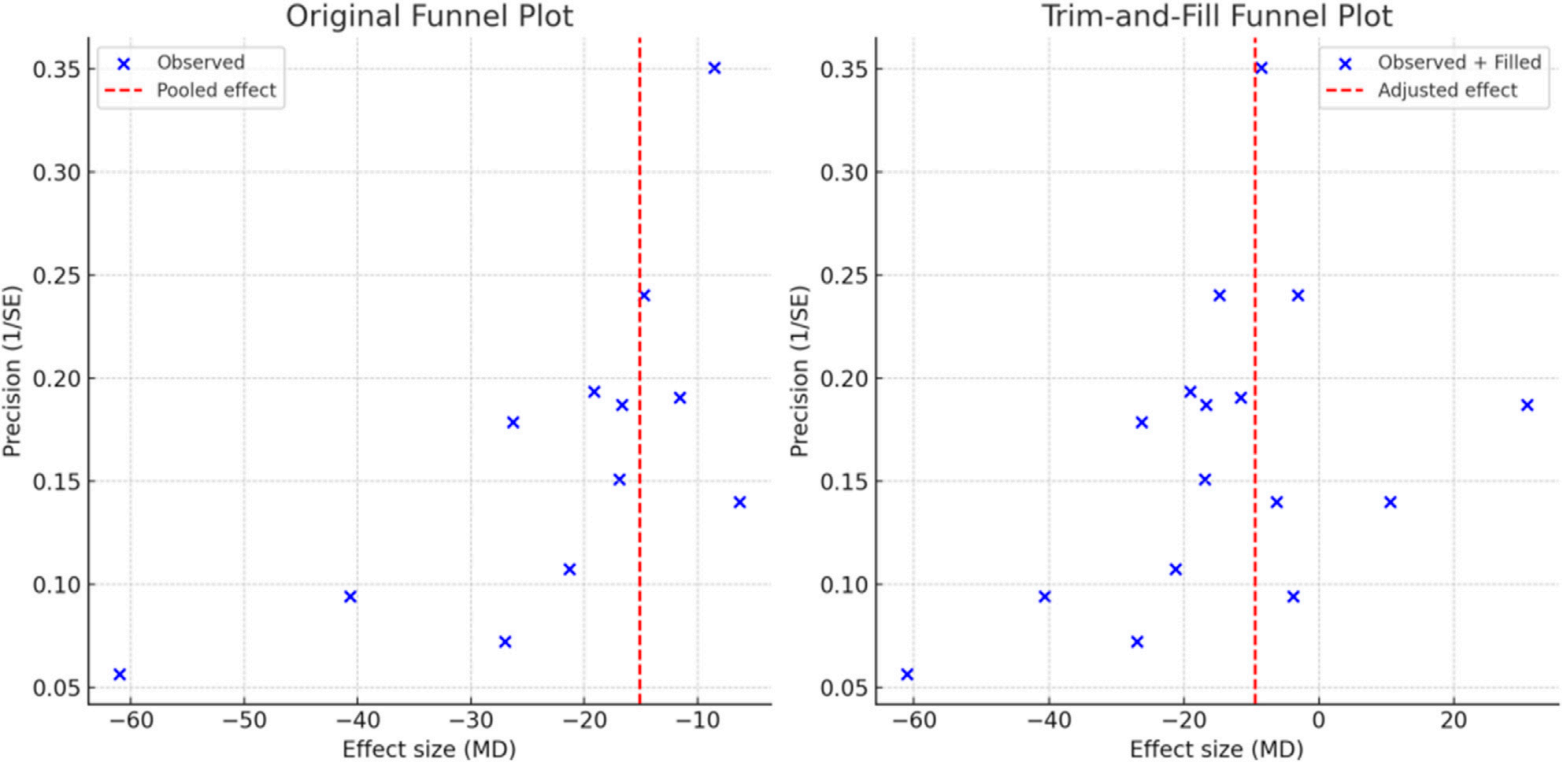
Forest plot of the meta-analysis of kidney function indicators.

Twelve studies (Lin et al., 2014; Zhang et al., 2016; Jiang et al., 2017; Luo et al., 2018; Zhang M. et al., 2019; Li et al., 2019; Zhu and Wu; Zhao, 2020; Yu, 2021; Huang et al., 2021; Song et al., 2022; Lin et al., 2022) reported data on serum creatinine, with the majority also reporting blood urea nitrogen. Meta-analysis showed that Xuebijing injection significantly reduced serum creatinine (p < 0.00001) and blood urea nitrogen (p < 0.00001) compared to control treatments. Additionally, several studies demonstrated a significant increase in urine volume following Xuebijing injection (p < 0.00001). Considerable heterogeneity was observed for serum creatinine and urine volume outcomes, whereas blood urea nitrogen showed low heterogeneity (Figure 4).

### 3.6 Inflammatory cytokines

**FIGURE 5:**
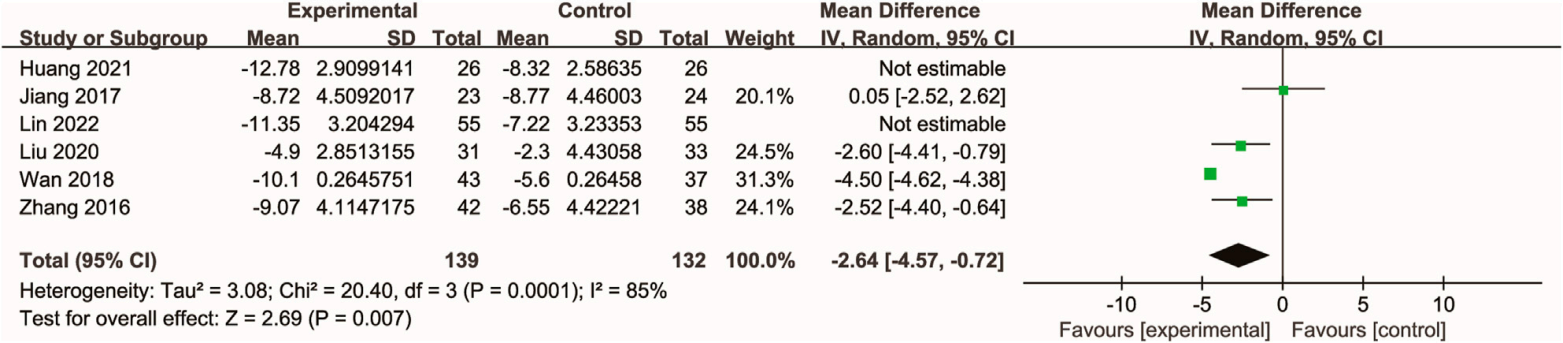
Forest plot of the meta-analysis of inflammatory cytokines.

Fourteen studies (Tang et al., 2014; Lin et al., 2014; Zhang et al., 2016; Yang, 2016; Jiang et al., 2017; Luo et al., 2018; Zhang S. Z. et al., 2019; Zhang M. et al., 2019; Li et al., 2019; Huang, 2019; Zhu and Wu; Zhao, 2020; Song et al., 2022; Lin et al., 2022) evaluated the effects of Xuebijing injection on inflammatory cytokines. The meta-analysis showed that Xuebijing injection significantly reduced levels of IL-6, TNF-α, and IL-10 compared to controls (all p < 0.01). Significant heterogeneity was noted across these analyses (Figure 5).

### 3.7 Immune function

**FIGURE 6:**
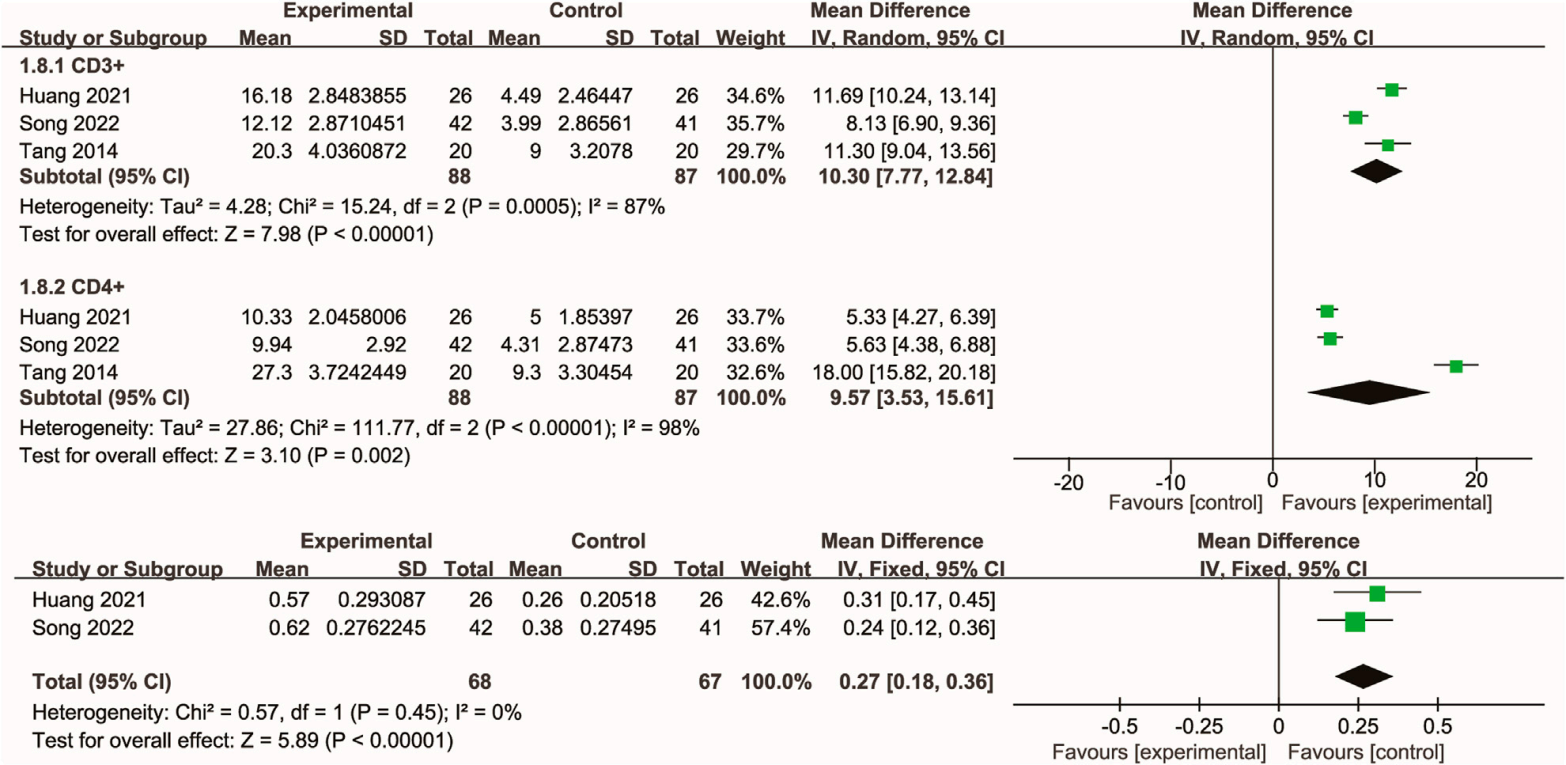
Forest plot of the meta-analysis of T-cell-based immune function.

Three studies (Tang et al., 2014; Huang et al., 2021; Song et al., 2022) evaluated the effects of Xuebijing injection on T-cell immune function. Meta-analysis indicated that Xuebijing injection significantly increased the percentages of CD3^+^ and CD4^+^ T cells, as well as the CD4+/CD8+ ratio, compared with control treatments (all p < 0.01). Substantial heterogeneity was observed for CD3^+^ and CD4^+^ T cell percentages, while the CD4+/CD8+ ratio showed low heterogeneity (Figure 6).

### 3.8 Severity of critical illness

**FIGURE 7:**
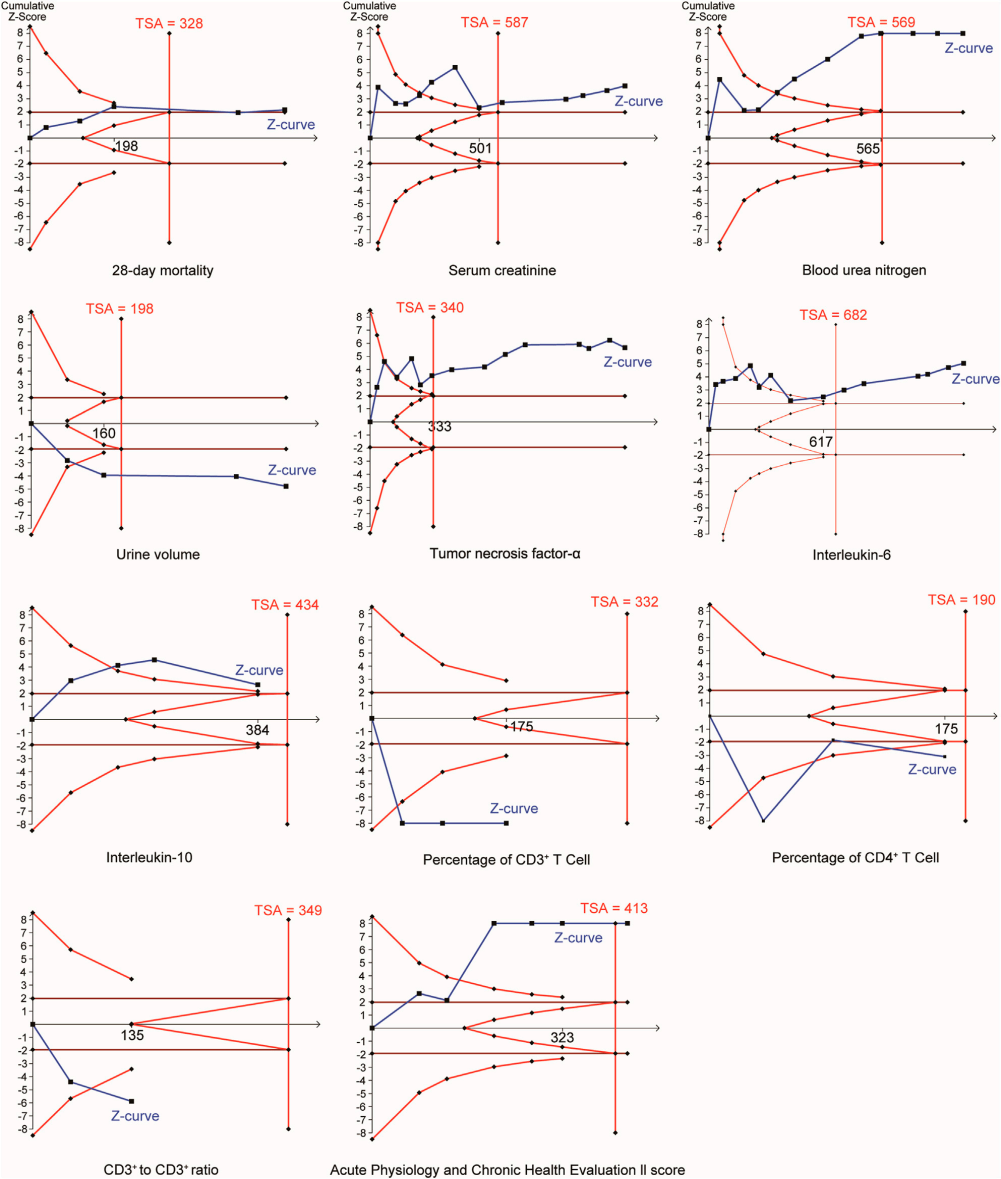
Forest plot of the meta-analysis of APACHE II scores.

Six studies (Zhang et al., 2016; Jiang et al., 2017; Wan et al., 2018; Liu et al., 2020; Huang et al., 2021; Lin et al., 2022) assessed the severity of SA-AKI using the APACHE II score. Meta-analysis showed that Xuebijing injection significantly reduced the APACHE II score compared with control treatments (p < 0.0001), although signifcant heterogeneity was observed across these studies (Figure 7).

### 3.9 Subgroup analysis

According to the subgroup analysis, 200 mL/d of Xuebijing injection resulted in a significantly greater inhibition of TNF-α levels compared with 100 mL/d (MD -0.71 ng/ml vs. −1.48 ng/ml; interaction p = 0.03). Additionally, patients with an average age of ≤50 years had a significantly greater reduction in IL-6 levels than those >50 years (MD -39.15 ng/ml vs. −14.40 ng/ml; P = 0.03). No significant subgroup difference was found among the other subgroup comparisons (Supplementary Table S3).

### 3.10 Sensitivity analysis

In the sensitivity analysis excluding RCTs with an overall high risk of bias, all the outcome estimates did not show directional changes (Supplementary Table S4).

### 3.11 Publication bias

**FIGURE 8:**
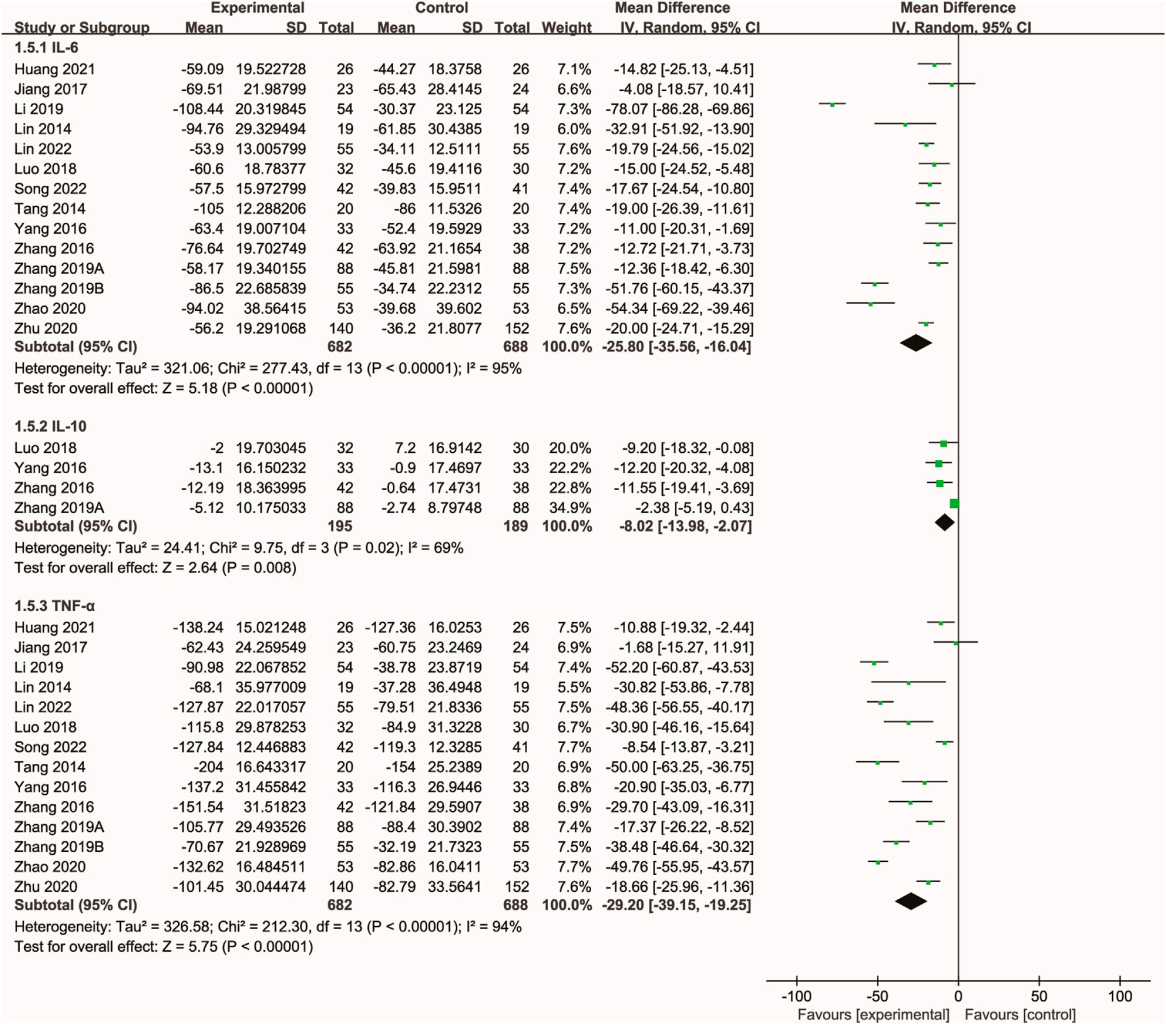
Funnel plot and trim-and-fill analysis of serum creatinine.

Four outcomes (serum creatinine, blood urea nitrogen, TNF-α, and IL-6) involved at least 10 RCTs and met the criteria for the detection of publication bias. As shown in Supplementary Figure S6, the funnel plot for serum creatinine exhibited asymmetry, which was corroborated by a significant Egger’s test result (p < 0.001), suggesting the presence of significant publication bias for this outcome. Trim-and-fill analysis was subsequently conducted, and after imputing hypothetically missing symmetric studies, the adjusted effect size for serum creatinine remained consistent with the original meta-analysis (p < 0.001), suggesting that the overall conclusion was robust despite potential bias. No significant publication bias was detected for the other outcomes. See Figure 8 for details.

### 3.12 TSA

**FIGURE 9:**
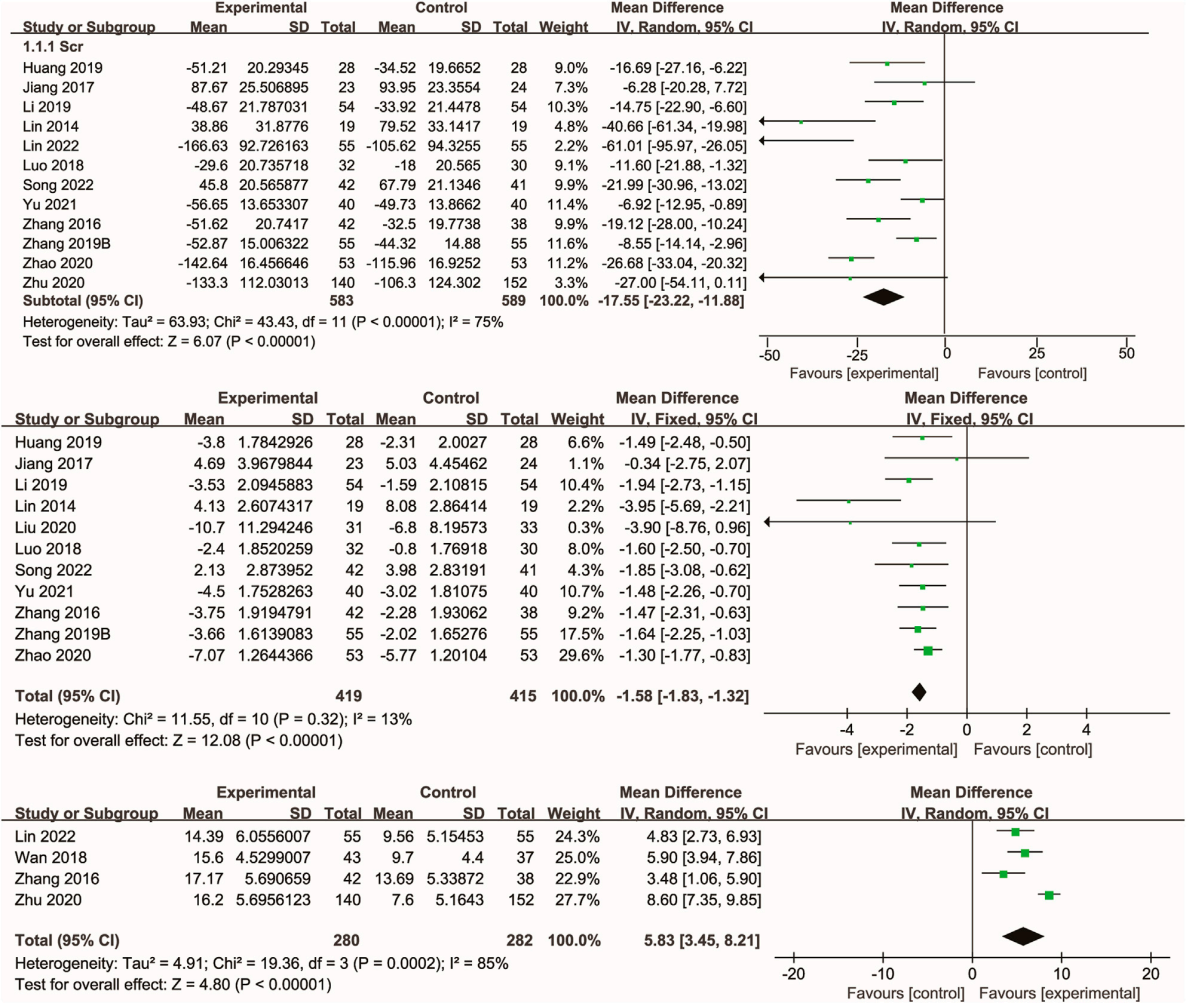
Results of the trial sequential analyses.

In the TSA (Figure 9), the Z curves of all outcomes crossed the TSA threshold, confirming that the significant between-group differences for these outcomes were not attributable to false-positive errors.

### 3.13 Quality of evidence

According to the GRADE assessment, the evidence for 28-day mortality and blood urea nitrogen was rated as moderate quality, with limitations primarily arising from risk of bias. The results for urine volume, CD4+/CD8+ ratio, and APACHE II score were classified as low-quality evidence due to concerns regarding heterogeneity. The remaining six outcomes were rated as very low-quality evidence due to limitations in multiple GRADE domains (Supplementary Table S5).

### 3.14 Safety

Two RCTs (Zhu and Wu, 2020; Yu, 2021) reported adverse events in the Xuebijing injection group, including 21 cases of pruritus, 13 cases of nausea and vomiting, 2 cases of dizziness, and 2 cases of hypotension, resulting in a total incidence of adverse events of 1.96%. In the control group, two cases of hypotension were documented. No treatment-related serious adverse events, such as respiratory depression or shock, were reported. The remaining RCTs did not report safety-related data.

## 4 Discussion

SA-AKI not only significantly increases the length of hospital stay, medical costs, and mortality but also raises the risk of post-discharge complications, including cardiovascular events and chronic kidney disease (Luo et al., 2022). Traditional Chinese medicine injections, known for their rapid onset of action, are suitable for critical care settings and offer a promising therapeutic option for SA-AKI. Although a previous meta-analysis investigated the use of Xuebijing injection for severe sepsis (Zhong et al., 2022), it had notable limitations: the literature search was incomplete (only seven studies were included), key methodological elements such as TSA and GRADE evaluation were lacking, and it did not specifically address kidney injury. In contrast, our systematic review included a total of 18 RCTs that specifically focused on SA-AKI and performed a comprehensive assessment that included critical care outcomes, such as the APACHE II score and inflammatory cytokines. The findings demonstrate that Xuebijing injection significantly reduced 28-day mortality and increased kidney function, immune function, and APACHE II scores in patients with SA-AKI. It also had positive effects on reducing the levels of inflammatory cytokines. Beyond sepsis, a recent systematic review demonstrated that Xuebijing also improved pulmonary ventilation parameters in acute pancreatitis (Bin et al., 2025), underscoring its systemic immunomodulatory potential.

The treatment of sepsis mainly depends on three aspects: controlling the excessive release of inflammatory cytokines, regulating the host’s septic response, and stabilizing hemodynamics (Lelubre and Vincent, 2018). Pathophysiological studies have shown that septic patients with AKI exhibit more significant systemic inflammation (e.g., increased levels of TNF-α, IL-6, and IL-10) than those without AKI (Matejovic et al., 2017). This heightened inflammatory state can cause cellular inflammatory cytokine storms and immunosuppression, promote platelet activation and aggregation, and ultimately lead to inflammatory tissue damage (Fani et al., 2018). A study based on serum pharmacochemistry revealed that Salvia miltiorrhiza, a key component of Xuebijing injection, is rich in phenolic acids such as protocatechuic acid and salvianolic acids A, B, and C (Yuan et al., 2024). These metabolites significantly inhibit the expression and release of inflammatory cytokines (e.g., TNF-α, IL-1β, and IL-6) induced by podocyte injury, which helps improve renal insufficiency caused by podocyte dysfunction and prevents renal parenchymal damage. This mechanism may explain why the Xuebijing injection group achieved greater reductions in serum creatinine and blood urea nitrogen levels.

The kidney is rich in capillaries, and sepsis-mediated microcirculatory dysfunction can lead to hypoperfusion, hypooxygenation, and a high-lactate state within the kidney (Liu et al., 2021). Animal experiments revealed that have identified 12 bioactive metabolites derived from Xuebijing injection—such as hydroxysafflower yellow A, paeoniflorin, oxypaeoniflorin, paeonifloride, and danshensu hexa—that exhibit widespread systemic distribution (Cheng et al., 2024). These metabolites not only improve the function of regulatory T cells and promote the proliferation of CD4^+^ effector T cells but also reduce levels of inflammatory cytokines in both serum and major organs, alleviate kidney damage, and improve coagulation function and vasodilation (Shen et al., 2023; Ge et al., 2023; Zhang and Wei, 2020). Comparable evidence from other flavonoids supports this notion; a meta-analysis indicated that quercetin alleviates AKI in animal models via anti-inflammatory and antioxidant mechanisms (Zeng et al., 2023). The observed increase in urine volume in the Xuebijing injection group indirectly affirms the drug’s beneficial effect on restoring renal perfusion. In addition, Xuebijing injection was shown to increase the proportion of Th1 cells and increase the percentage of CD4^+^CD25^+^ cells, thereby regulating the immune response and reversing sepsis-associated immunosuppression (Li C. et al., 2021). This timely rescue of residual renal function also contributed to the improvement in APACHE II scores in the Xuebijing injection group.

Subgroup analysis suggested that a 200 mL/day dose of Xuebijing injection had a greater effect on reducing inflammatory cytokine levels compared with a 100 mL/day regimen. This finding is similar to those of previous studies. For example, Wang et al. (2023) reported that high-dose Xuebijing injection significantly improved the coagulation status, C-reactive protein level, and lactic acid level in patients with sepsis compared with low-dose treatment. Therefore, for SA-AKI patients who exhibit adequate tolerance, we recommend a dosage of 100 ml per administration to potentially achieve better efficacy.

Only two RCTs provided safety data. Adverse events in the Xuebijing injection group are mainly mild rash and nausea (Zhu and Wu, 2020; Yu, 2021). However, owing to the limited sample size, it cannot be directly concluded that Xuebijing injection has good tolerance. A broader safety evaluation, which incorporated data from 211 publications involving 46,384 patients receiving Xuebijing injection, documented 423 adverse reactions (Li Q. et al., 2021). More than half of these affected the skin (151 cases), cardiovascular system (68 cases), or gastrointestinal system (65 cases); common manifestations included rash, pruritus, palpitations, hypotension, nausea, vomiting, and diarrhea. These reactions were generally mild and resolved after drug discontinuation or symptomatic treatment. Studies suggest that safflower yellow A and ligustrazine in Xuebijing injection may act as allergens. They could stimulate the production of antibodies or sensitize lymphocytes, potentially triggering allergic reactions upon re-exposure (Liu et al., 2019). According to the “Basic Principles of Clinical Use of Chinese Medicine Injections” promulgated by the Chinese government, factors such as infusion rate, whether the intravenous line was flushed, history of allergies, age (particularly 60 years and older), and concomitant use with other TCM injections, antibacterial agents, or immune-enhancing drugs may be associated with an increased risk of adverse reactions (Sun, 2017). Therefore, we recommend strict adherence to operational procedures during the clinical administration of Xuebijing injection, including a slow infusion rate and close monitoring for high-risk populations.

This review has several limitations. First, none of the included studies reported the specific procedures for allocation concealment and blinding, which represents a major source of risk of bias and is one of the main reasons for downgrading the certainty of evidence in the GRADE assessment. Although the sensitivity analysis excluding studies with an overall high risk of bias did not reveal important differences—suggesting that the risk of bias had a limited impact on the conclusions—the lack of these methodological safeguards may still lead to overestimation of treatment effects. For example, studies without allocation concealment and blinding have been shown to be more likely to exaggerate treatment benefits (Savović et al., 2012). Therefore, the methodological weaknesses associated with risk of bias should be taken into consideration when interpreting the findings. Second, T-cell subsets are clinically important indicators of autoimmune status in patients with SA-AKI, but the number of studies and sample size were small, and the accuracy of the results was affected. Third, although subgroup analyses stratified by age, treatment dose, and treatment duration explaining some heterogeneity, substantial unexplained heterogeneity remained across several outcomes, which limits the robustness of the pooled estimates. Some prognostic factors, such as the type and severity of infection, baseline renal function, and comorbidities (e.g., diabetes or cardiovascular disease), may contribute to heterogeneity; however, due to the limited number of studies reporting these subgroup data, we were unable to perform further analyses. Fourth, evidence of publication bias was detected for serum creatinine, which reduces confidence in the observed renal benefits of Xuebijing injection. Nevertheless, the results of the trim-and-fill analysis were consistent with the direction of the primary analysis, implying that unpublished studies would not substantially alter the conclusions. Fifth, all included RCTs were conducted in China, raising concerns regarding external validity and generalizability. Variations in healthcare systems, clinical practice patterns, patient demographics, and ancillary treatment strategies across regions may limit the applicability of our findings to non-Chinese populations. Therefore, further multicenter trials across diverse populations are necessary before the applicability of Xuebijing injeciton can be confirmed internationally.

## 5 Conclusion

Xuebijing injection, when used as an adjunctive therapy for SA-AKI, may improve renal function, immune responses, and inflammatory status, and ultimately reduce mortality. However, as the certainty of evidence is limited by risk of bias, heterogeneity, and publication bias, high-quality, large-scale, double-blind RCTs are needed to validate these findings.

## Data Availability

The original contributions presented in the study are included in the article/Supplementary Material, further inquiries can be directed to the corresponding authors.

## Abbreviations

SA-AKI, sepsis-associated acute kidney injury; TNF-α, tumor necrosis factor-alpha; IL-6, interleukin-6; IL-10, interleukin-10; APACHE II, Acute Physiology and Chronic Health Evaluation II; CI, confidence interval; GRADE, Grading of Recommendation, Assessment, Development and Evaluation; RCT, randomized controlled trial; RR, relative risk; TSA, trial sequential analysis.
