# Supplementary material for: Comparative efficacy and safety of Xuebijing injection as adjuvant therapy in sepsis-associated acute kidney injury: a systematic review and meta-analysis
Source: Front Pharmacol. 2025 Oct 21;16:1643557. doi: 10.3389/fphar.2025.1643557 (PMC12583064; doi:10.3389/fphar.2025.1643557)
Supplement: Supplementary file 1 [file Supplementaryfile1.docx]

**Supplemental file**

Contents

[Table S.1. Search strategies. 2](#_Toc175495490)

[Table S.2. List of main excluded studies and the reasons for exclusion. 3](#_Toc175495491)

[Table S.3. Results of subgroup analyses. 4](#_Toc175495493)

[Table S.4. Results of sensitivity analyses. 5](#_Toc175495494)

[Table S.5. Results of the GRADE assessment of the quality of evidence. 6](#_Toc175495495)

Figure S.6. Funnel plots and results of Egger's tests 8

Table S.1. Search strategies.

| PubMed | (xuebijing[Title/Abstract] OR (Xue Bi Jing[Title/Abstract] OR "Chinese Drugs, Plant"[Title/Abstract] OR "Plant Extracts, Chinese"[Title/Abstract] OR Chinese Plant Extracts[Title/Abstract]) AND (Sepsis[Title/Abstract] OR Bloodstream Infection[Title/Abstract] OR Septicemia[Title/Abstract] OR (Blood Poisoning[Title/Abstract] OR Severe Sepsis[Title/Abstract] OR Pyemia[Title/Abstract]) AND (Acute Kidney Injury[Title/Abstract] OR Acute Renal Injury[Title/Abstract] OR Acute Kidney Failures[Title/Abstract] OR Acute Renal Failure[Title/Abstract]) |
| --- | --- |
| EMBASE | ( 'xue bi jing injection'/exp OR 'xue-bi-jing'/exp OR 'xue-bi-jing injection' OR 'xue-bi-jing-zhu-she' OR 'xuebijing injection'/exp) AND ( 'abdominal sepsis' OR 'focal sepsis'/exp OR 'intraabdominal sepsis'/exp OR 'sepsis syndrome'/exp OR 'sepsis syndrome' OR 'septic disease'/exp OR 'septic disease' OR 'sepsis'/exp OR 'sepsis') AND ( 'acute kidney injury' OR 'acute kidney insufficiency'/exp OR 'acute renal failure'/exp OR 'acute renal insufficiency'/exp OR 'kidney acute failure'/exp OR 'kidney failure, acute'/exp OR 'kidney insufficiency, acute'/exp OR 'renal insufficiency, acute'/exp OR 'acute kidney failure'/exp) |
| CNKI | TKA=(血必净+血必净注射剂+中药注射剂)*(脓毒症+败血症+脓毒性休克)*(急性肾病+急性肾脏病+急性肾衰竭+急性肾损伤+急性肾功能损伤+急性肾功能不全+急性肾损害+急性肾脏损害+AKI) |
| Wanfang | 题名或关键词:(“血必净”) and (“脓毒症”) and (“急性肾病”or “急性肾脏病”or “急性肾衰竭”or “急性肾损伤”or “急性肾功能损伤”or “急性肾功能不全”or “急性肾损害”or “急性肾脏损害”or “AKI”) not (“动物”or “大鼠”or “小鼠” ) |
| VIP | U=(血必净) AND (脓毒症) AND (急性肾病 OR 急性肾脏病 OR 急性肾衰竭 OR 急性肾损伤 OR 急性肾功能损伤 OR 急性肾功能不全 OR 急性肾损伤 OR 急性肾损害 OR 急性肾脏损害 OR AKI) not (动物 OR 大鼠 OR 小鼠) |
| Chinese Biomedical Literature database | ("急性肾损伤"[常用字段:智能] OR "急性肾功能衰竭"[常用字段:智能] OR "急性肾脏功能衰竭"[常用字段:智能] OR "急性肾脏功能不全"[常用字段:智能]) AND ("脓毒症"[常用字段:智能] OR "脓毒血症"[常用字段:智能] OR "脓血症"[常用字段:智能] OR "败血病"[常用字段:智能] OR "血液中毒"[常用字段:智能] OR "严重败血症"[常用字段:智能]) AND ("血必净"[常用字段:智能]) |

Table S.2. List of the main excluded studies and the reasons for exclusion.

| ***Not randomized controlled trial*** |
| --- |
| 1. Wang J, Luo C, Luo M, Zhou S, Kuang G. Targets and Mechanisms of Xuebijing in the Treatment of Acute Kidney Injury Associated with Sepsis: A Network Pharmacology-based Study. Curr Comput Aided Drug Des. 2024;20(6):752-763.  2. Dong Q, Huang Y, Yao M, et al. Analysis of the Research Status of Traditional Chinese Medicine Treatment for Sepsis Based on CiteSpace[J]. Hainan Medical Journal, 2021, 32(07): 930-935.  3. Jiang Y, Zou L, Liu S, Liu X, Chen F, Liu X, Zhu Y. GC/MS-based metabonomics approach reveals effects of Xuebijing injection in CLP induced septic rats. Biomed Pharmacother. 2019 Sep;117:109163. doi: 10.1016/j.biopha.2019.109163. Epub 2019 Jun 22. PMID: 31238257. |
| ***Ineligible patients*** |
| 1. Liang W, Fu B, Zhao X, et al. Effect of Xuebijing injection combined with low molecular weight heparin on the levels of Pro-C5a and heparanase in elderly patients with acute respiratory distress syndrome[J]. Journal of Practical Medicine, 2022, 38(14): 1814-1818.  2. Zhang C, Zhang H, Wang S, et al. The efficacy of Xuebijing combined with piperacillin/tazobactam in the treatment of severe pneumonia in elderly patients[J]. Chinese Journal of Gerontology, 2022, 42(09): 2128-2131.  3. Peng F, Liu R, Niu J, et al. Effects of Xuebijing Injection in combination with routine symptomatic interventions on Th1/Th2 balance and the expressions of TLR2 and TLR4 in patients with severe intra - abdominal infection [J]. Journal of Chinese Medicinal Materials, 2020, 43(9): 2287 - 2290.  4. Meng X, Yang S, Cao Y, et al. Observation on the clinical efficacy of Xuebijing Injection in patients with bacterial liver abscess complicated with sepsis [J]. Chinese Journal of Critical Care Medicine, 2019, 39(11): 1050 - 1052.  5. Yuan W, Chen R, Li X. Effects of Shenfu Injection combined with Xuebijing Injection on circulation and capillary refill time in patients with septic cardiomyopathy [J]. Journal of Zunyi Medical University, 2024, 47(1): 70 - 75. |
| ***Ineligible intervention*** |
| 1. Nie Y, Chen H. Clinical observation on Xuebijing Injection combined with No.1 Traditional Chinese Medicine Enema Recipe in the treatment of early mild sepsis - associated acute kidney injury [J]. Journal of Practical Traditional Chinese Medicine, 2019, 35(1): 27 - 28.  2. Wang X, Zhang Y, Gong S. Clinical efficacy of Shenmai Injection and Xuebijing Injection in the treatment of sepsis and their effects on immune function [J]. Jilin Medical Journal, 2024, 45(8): 1900 - 1902.  3. Jiang Y, Hu H, Gu W. Observation on the clinical efficacy of Baihu Decoction combined with Xuebijing Injection in the treatment of sepsis (syndrome of intense internal toxin - heat) [J]. Proceeding of Clinical Medicine, 2024, 33(5): 327 - 330.  4. Chen S, Tang X, Hu B, et al. Analysis of the clinical efficacy of Shenfu Injection and Xuebijing Injection in the treatment of sepsis patients [J]. Practical Clinical Journal of Integrated Traditional Chinese and Western Medicine, 2024, 24(4): 46 - 48 + 124. |

Table S.3. Results of the subgroup analysis.

| **Outcome** | **Factor** | **Type of subgroup** | **No. of studies** | **Effect size (95% CI)** | **I^2^** | **Interaction p value** |
| --- | --- | --- | --- | --- | --- | --- |
| Serum creatinine (μmol/L) | All studies |  | 12 | MD-17.55 (-23.22, -11.88) | 75% |  |
|  | Age | ≤ 50 years old | 7 | MD -12.90 (-24.64, -1.16) | 94% | 0.52 |
|  |  | > 50 years old | 5 | MD -18.07 (-28.79, -7.35) | 89% |  |
|  | Dose of XBJ | 50 ml | 5 | MD -0.70(-1.08, -0.31) | 72% | 0.61 |
|  |  | 100 ml | 7 | MD -0.52(-1.07, 0.02) | 91% |  |
|  | Course of treatment | ≤ 7 days | 10 | MD -17.78(-24.63, -10.93) | 79% | 0.95 |
|  |  | > 7 days | 2 | MD-18.10(-24.87, -11.33) | 0% |  |
| Tumor necrosis factor-alpha (ng/ml) | All studies |  | 14 | MD -29.20 (-39.15, -19.25) | 94% |  |
|  | Age | ≤ 50 years old | 7 | MD-34.78 (-52.42, -17.14) | 97% | 0.32 |
|  |  | > 50 years old | 7 | MD -24.14 (-35.74, -12.53) | 88% |  |
|  | Course of treatment | ≤ 7 days | 11 | MD-28.05 (-39.61, -16.48) | 95% | 0.59 |
|  |  | > 7 days | 3 | MD -33.66 (-50.57, -16,74) | 78% |  |
|  | Dose of XBJ | 50 ml | 5 | MD -0.71 (-0.89, -0.54) | 0% | 0.03 |
|  |  | 100 ml | 9 | MD -1.48 (-2.13, -0.83) | 94% |  |
| Interleukin-6 (ng/ml) | All studies |  | 14 | MD -25.80 (-35.56, -16.04) | 95% |  |
|  | Age | ≤ 50 years old | 7 | MD -39.15 (-60.70, -17.61) | 97% | 0.03 |
|  |  | > 50 years old | 7 | MD-14.40 (-18.85, -9.95) | 61% |  |
|  | Course of treatment | ≤ 7 days | 11 | MD -28.35(-40.53, -16.16) | 96% | 0.05 |
|  |  | > 7 days | 3 | MD -14.94(-19.92, -9.97) | 4% |  |
|  | Dose of XBJ | 50 ml | 6 | MD -16.72(-21.90, -11.54) | 41% | 0.12 |
|  |  | 100 ml | 8 | MD -29.30(-44.17, -14.44) | 97% |  |

Table S.4. Results of the sensitivity analysis.

| **Outcome** | **Analysis** | **No. of patients** | | **WMD/SMD**  **(95% CI)** | **Heterogeneity** |
| --- | --- | --- | --- | --- | --- |
|  |  | **XBJ** | **Control** |  |  |
| Serum creatinine (μmol/L) | Main analysis | 583 | 589 | -17.55 (-23.22, -11.88) | I² = 75%, P < 0.00001 |
|  | Excluding trials with high risk of bias | 327 | 333 | -5.93 (-9.02, -2.85) | I² = 60%, P < 0.00001 |
| Blood urea nitrogen (mmol/L) | Main analysis | 419 | 415 | -9.60 (-14.96, -4.25) | I² = 13%, P < 0.00001 |
|  | Excluding trials with high risk of bias | 287 | 285 | -1.64 (-1.99, -1.29) | I² = 0%, P < 0.00001 |
| Tumor necrosis factor-alpha (ng/ml) | Main analysis | 682 | 688 | -29.20 (-39.15, -19.25) | I² = 94%, P < 0.00001 |
|  | Excluding trials with high risk of bias | 265 | 258 | -5.26 (-10.98, 0.46) | I² = 95%, P =0.0002 |
| Interleukin-6 (ng/ml) | Main analysis | 682 | 688 | -25.80 (-35.56, -16.04) | I² = 95%, P < 0.00001 |
|  | Excluding trials with high risk of bias | 357 | 353 | -25.00 (-42.34, -7.66) | I² = 97%, P = 0.005 |
| Acute Physiology and Chronic Health Evaluation II | Main analysis | 220 | 213 | -3.12 (-4.51, -1.73) | I² = 76%, P < 0.00001 |
|  | Excluding trials with high risk of bias | 330 | 320 | -2.64 (-4.57, -0.72) | I² = 85%, P =0.007 |

Table S.5. Results of the GRADE assessment of the quality of evidence

| **Quality assessment** | | | | | | | **No of patients** | | **Effect** | | **Quality** | **Importance** |  |
| --- | --- | --- | --- | --- | --- | --- | --- | --- | --- | --- | --- | --- | --- |
|  |  |  |  |  |  |  |  |  |  |  |  |  |  |
| **No of studies** | **Design** | **Risk of bias** | **Inconsistency** | **Indirectness** | **Imprecision** | **publication bias** | **experimental** | **Control** | **Relative (95% CI)** | **Absolute (95% CI)** |  |  |  |
| **28-day mortality rate** | | | | | | | | | | | | |  |
| 5 | randomized trials | serious^1^ | no serious inconsistency | no serious indirectness | no serious imprecision | Not detected | 115/299  (38.5%) | 144/301  (47.8%) | RR 0.83 (0.68 to 1.02) | 81 fewer per 1000 (from 153 fewer to 10 more) | ⊕⊕⊕○ MODERATE | CRITICAL |  |
|  |  |  |  |  |  |  |  | 43.2% |  | 73 fewer per 1000 (from 138 fewer to 9 more) |  |  |  |
| **Serum creatinine (Better indicated by lower values)** | | | | | | | | | | | | |  |
| 12 | randomized trials | serious^1^ | serious^2^ | no serious indirectness | no serious imprecision | Not detected | 583 | 589 | - | MD 16.2 lower (24.86 to 7.54 lower) | ⊕○○○ VERY LOW | CRITICAL |  |
| **Blood urea nitrogen (Better indicated by lower values)** | | | | | | | | | | | | |  |
| 11 | randomized trials | serious^1^ | No serious inconsistency | no serious indirectness | no serious imprecision | Not detected | 419 | 415 | - | MD 1.61 lower (1.97 to 1.25 lower) | ⊕⊕⊕O MODERATE | CRITICAL |  |
| **Urine volume (Better indicated by lower values)** | | | | | | | | | | | | |  |
| 4 | Randomized trials | serious^1^ | serious^2^ | no serious indirectness | no serious imprecision | Not detected | 280 | 282 | - | MD 5.83 higher (3.45 to 8.21 higher) | ⊕⊕○○ LOW | CRITICAL |  |
| **Tumor Necrosis Factor-α (Better indicated by lower values)** | | | | | | | | | | | | |  |
| 12 | randomized trials | serious^1^ | very serious^2^ | no serious indirectness | no serious imprecision | Not detected | 614 | 621 | - | MD 32.5 lower (42.09 to 22.91 lower) | ⊕○○○ VERY LOW | IMPORTANT |  |
| **Acute Physiology and Chronic Health Evaluation II score (Better indicated by lower values)** | | | | | | | | | | | | |  |
| 6 | Randomized trials | serious^1^ | serious^2^ | no serious indirectness | no serious imprecision | Not detected | 220 | 213 | - | MD 3.41 lower (4.46 to 2.37 lower) | ⊕⊕○○ LOW | IMPORTANT |  |
| **Interleukin-10 (Better indicated by lower values)** | | | | | | | | | | | | |  |
| 4 | Randomized trials | serious^1^ | serious^2^ | no serious indirectness | Serious^3^ | Not detected | 195 | 189 | - | MD 8.02 lower (13.98 to 2.07 lower) | ⊕○○○ VERY LOW | IMPORTANT |  |
| **Interleukin-6 (Better indicated by lower values)** | | | | | | | | | | | | |  |
| 14 | Randomized trials | serious^1^ | very serious^2^ | no serious indirectness | no serious imprecision | Not detected | 682 | 688 | - | MD 25.26 lower (35.14 to 15.39 lower) | ⊕○○○ VERY LOW | IMPORTANT |  |
| **Percentage of CD 4+ T cell (Better indicated by lower values)** | | | | | | | | | | | | |  |
| 3 | Randomized trials | serious^1^ | serious^2^ | no serious indirectness | Serious^3^ | Not detected | 88 | 87 | - | MD 10.3 higher (7.77 to 12.84 higher) | ⊕○○○ VERY LOW | IMPORTANT |  |
| **Percentage of CD 8+ T cell (Better indicated by lower values)** | | | | | | | | | | | | |  |
| 3 | Randomized trials | serious^1^ | serious^2^ | no serious indirectness | Serious^3^ | Not detected | 88 | 87 | - | MD 9.57 higher (3.53 to 15.61 higher) | ⊕○○○ VERY LOW | IMPORTANT |  |
| **CD 4+/CD 8+ ratio (Better indicated by lower values)** | | | | | | | | | | | | |  |
| 2 | Randomized trials | serious^1^ | no serious inconsistency | no serious indirectness | Serious^3^ | Not detected | 68 | 67 | - | MD 0.27 higher (0.18 to 0.36 higher) | ⊕⊕○○ LOW | IMPORTANT |  |

^1^ No studies with overall low risk of bias were available, so the quality of evidence for all outcomes was downgraded by one level for risk of bias; for outcomes with a result changed in direction after excluding studies with overall high risk of bias, an additional downgrade by one level was applied.

^2^ If I^2^ < 50%, there was no downgrading. If 50% ≤ I^2^ ≤ 75%, the quality of evidence was downgraded by one level if any point estimate of primary studies was at the opposite site, or there was no downgrading. If I^2^ > 75%, the quality of evidence was downgraded by two levels if any point estimate of primary studies was at the opposite site, or the quality was downgraded by one level.

^3^ Because the Z curves of all the outcomes passed through the TSA threshold, there was no downgrading.

^4^ If publication bias was identified with a funnel plot or Egger's test, the quality of evidence was downgraded by one level. For other situations, including an insufficient number of included RCTs to detect publication bias, the quality of evidence was not downgraded.

^5^ All outcomes were a direct reflection of efficacy, so there was no downgrading due to indirectness.

Abbreviations: CI = confidence interval; MD = mean difference; NR = not reported; RCT= randomized controlled trial; RD = risk difference; RR = risk ratio.


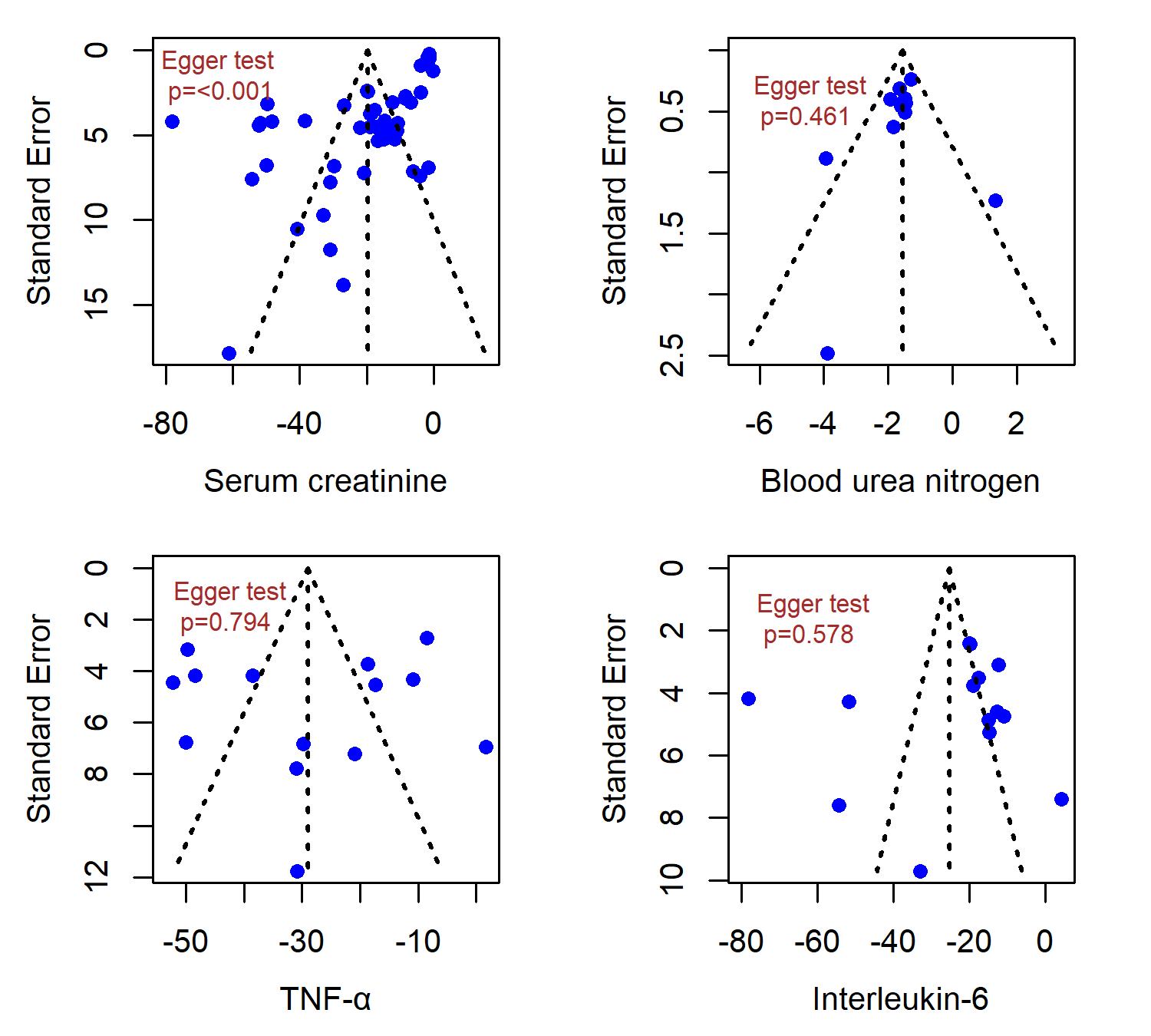


**Figure S.6 Funnel plots and results of Egger's tests.**
